# A Meta-Analysis of the Efficacy of Interferon Monotherapy or Combined with Different Nucleos(t)ide Analogues for Chronic Hepatitis B

**DOI:** 10.3390/ijerph13070730

**Published:** 2016-07-21

**Authors:** Jialing Zhou, Xiaoning Wu, Wei Wei, Hong You, Jidong Jia, Yuanyuan Kong

**Affiliations:** 1 Liver Research Center, Beijing Friendship Hospital, Capital Medical University, Beijing 100050, China; zhoujialing11@126.com (J.Z.); wuxiaoningbs@126.com (X.W.); youhong30@sina.com (H.Y.); jia_jd@ccmu.edu.cn (J.J.); 2 Beijing Key Laboratory of Translational Medicine in Liver Cirrhosis, Beijing Friendship Hospital, Capital Medical University, Beijing 100050, China; 3 Clinical Epidemiology and Evidence-Based Medicine Unit, National Clinical Research Center for Digestive Diseases, Beijing 100050, China; vivi_0306@126.com

**Keywords:** chronic hepatitis B, nucleos(t)ide analogues, interferon, combination therapy, meta-analysis

## Abstract

*Background:* The aim of the present study was to compare the efficacy of interferon (IFN) with or without different nucleos(t)ide analogues (NAs). *Methods:* The PubMed, Wan Fang and CNKI databases were searched to identify relevant trials up to May 2015. Meta-analysis was performed with Review Manager 5.0. The stability and reliability were evaluated by publication bias tests. *Results:* Fifty-six studies fulfilled the criteria for the meta-analysis. Compared with IFN monotherapy, combination therapy were superior in HBV DNA undetectable rate (Risk Ratio (RR) = 1.55, 95% confidence interval (CI): 1.44–1.66, *p* < 0.00001), HBeAg and HBsAg loss rate (RR = 1.38, 95% CI: 1.22–1.56, *p* < 0.00001; RR = 1.69, 95% CI: 1.03–2.78, *p* = 0.04, respectively) at the end of week 48 treatment. Sub-analysis showed the RRs of virological response for entecavir (ETV), adefovir (ADV), and lamivudine (LAM) were 1.64, 1.61 and 1.52, respectively; RRs of HBeAg loss rate were 1.34, 1.71 and 1.34, respectively. However, at the end of follow-up, IFN plus NAs therapy was better than IFN monotherapy only in terms of HBV DNA undetectable rate (*p* = 0.0007). *Conclusions:* Combination therapy was better than IFN monotherapy in virological and serological responses at the end of treatment. After follow-up, only HBV DNA undetectable rate was superior for combination therapy.

## 1. Introduction

Chronic hepatitis B virus (HBV) infection is a severe burden on public health. Current consensus guidelines on CHB recommend either a finite course of PEG IFN which stimulates immune response against the virus [1,2] or NA treatment of indefinite duration which directly suppresses replication of HBV [3,4,5].

However, the efficacy of monotherapy with IFN or NAs has been unsatisfactory. NAs directly inhibit viral replication by targeting HBV DNA, while IFN is an important immuno-modulator that interacts with the adaptive and innate immune responses, but also has limited direct antiviral effect on HBV [6,7]. Theoretically, combining NAs and IFN, with their different mechanisms of action, is an attractive therapeutic regimen for treating CHB. Therefore, the combination therapies using NAs and IFN have been extensively studied with a rationale that they may have synergistic antiviral effects.

However, previous studies on the efficacy of IFN with or without NA combination showed controversial results [8,9,10,11,12,13]. Published meta-analysis mainly addressed the efficacy of one NA at the end of treatment and gave inconsistent efficacy results combined with IFN at the end of follow-up. Therefore, we carried out this meta-analysis to compare the efficacy between IFN monotherapy and IFN combined with different NAs (ETV, ADV and LAM) and with a special focus on the efficacy at the end of follow-up.

## 2. Materials and Methods

### 2.1. Search Strategy

Databases including PubMed, CNKI [14] and Wan Fang [15] were searched for relevant literature published up to 30 May 2015. The CNKI and Wan Fang databases are the largest and relatively authoritative electronic databases in China. Both of these Chinese databases are internet-based, and have been widely subscribed by many universities and academic institutions. 

The following searches were used: ((peginterferon OR peg interferon OR pegylated interferon OR peg-ifn OR peg ifn OR pegasys OR peginterferon) AND (nucleotide analogue$1 OR nucleotide analog$1 OR nucleoside analog$1 OR nucleoside analogue$1 OR NAs OR lamivudine OR Lam OR adefovir OR Adv OR entecavir OR ETV OR tenofovir OR TDF) AND (chronic hepatitis b OR CHB OR HBV OR hepatitis b virus OR hepatitis b) OR (cirrhosis OR fibrosis)). The literature languages were limited to English and Chinese.

### 2.2. Inclusion and Exclusion Criteria

We included trials meeting the following criteria:

(i) randomized controlled trials including HBeAg-positive and/or negative adult CHB patients;
(ii) the trial drugs included IFN combination with NAs (LAM, ADV or ETV) and IFN mono-therapy; and
(iii) the therapy was administered for at least 24 weeks; and
(iv) initial combination therapy was needed.

The exclusion criteria were:

(i) inclusion of patients co-infected with human immune deficiency virus, hepatitis C virus, or hepatitis D virus;
(ii) the study did not evaluate efficacy.

### 2.3. Definitions of Efficacy

The virological and serological responses at the end of at least 24 weeks of follow-up were used as the primary efficacy endpoint. The secondary endpoints included virological and serological response at week 24 and 48 treatment respectively. In this meta-analysis, virological response was accessed by “undetectable” for HBV DNA, which defined as a level of HBV DNA < 400 copies per milliliter. Serological response was evaluated by HBeAg and HBsAg loss rates which were below the minimum detection limit (undetectable) [16,17].

### 2.4. Data Extraction

Two investigators independently conducted the literature search and screened the titles and abstracts of all collected studies. Then, a full text screening was used to ensure each met the inclusion criteria. Data extraction from each manuscript was also carried out independently.

### 2.5. Statistical Analysis

The meta-analysis was performed to assess differences in virological and serological efficacy between combination therapy and mono-therapy. Risk ratios (RRs) and 95% confidence intervals (CI) were reported. The statistical significance was set at *p* < 0.01. Fixed effect model was applied if there was no heterogeneity detected among trials [18]. The collected data were processed by the statistical software Review Manager, version 5.1 (The Nordic Cochrane Centre, The Cochrane Collaboration, Copenhagen, Denmark).

A funnel plot was applied to check publication bias. A roughly funnel-shaped distribution indicated absence of publication bias, showing the largest studies plotted near the average, and smaller studies spread evenly on both sides of the average. Deviation from this shape indicated publication bias.

The quality of all included RCTs was assessed using the Modified Jadad quality scale, which graded the quality of a study from 0 (lowest) to 7 (highest) by examining randomization, blinding, allocation concealment, and drop-out.

## 3. Results

### 3.1. Selection and Characteristics of Studies

**Figure 1:**
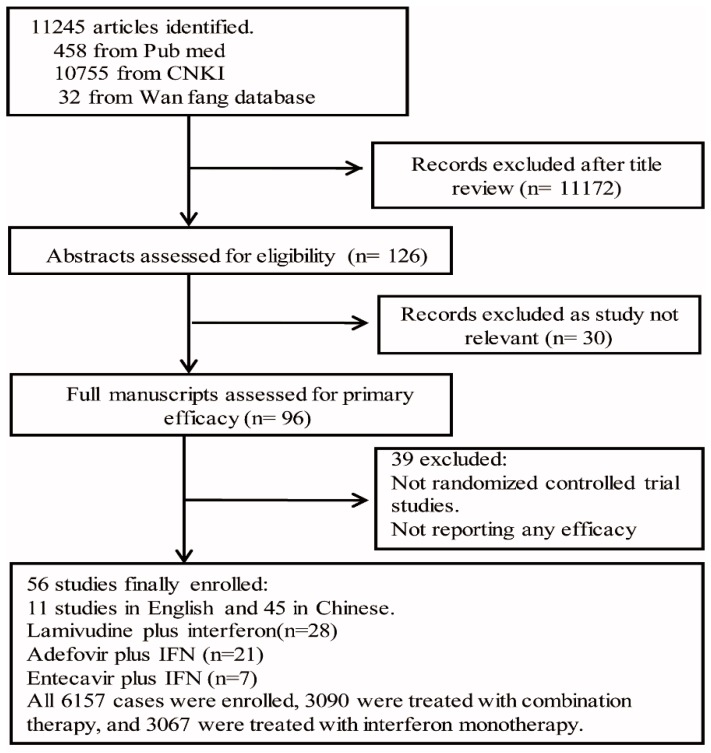
Flow chart of study selection and extraction.

A flow diagram detailing the selection of eligible studies is shown in Figure 1. Finally, a total of 56 trials were selected from 11,245 potentially eligible studies, all of which were published as full research papers (11 in English and 45 in Chinese) [8,9,10,11,12,16,17,19,20,21,22,23,24,25,26,27,28,29,30,31,32,33,34,35,36,37,38,39,40,41,42,43,44,45,46,47,48,49,50,51,52,53,54,55,56,57,58,59,60,61,62,63,64,65,66,67,68]. Combination therapies were used in the 56 trials, including IFN combined with LAM in 28 trials [8,9,10,11,12,16,17,19,20,21,22,23,24,25,26,27,28,29,30,31,32,33,34,35,36,37,38,39,40], ADV in 21 trials [41,42,43,44,45,46,47,48,49,50,51,52,53,54,55,56,57,58,59,60,61], and ETV in seven trials [62,63,64,65,66,67,68], respectively. The characteristics of studies included for this systematic review are listed in Supplementary Materials Table S1.

### 3.2. Better Efficacy of Combination Therapy in Virological and Serological Responses at Week 24 and 48 of Treatment

**Figure 2:**
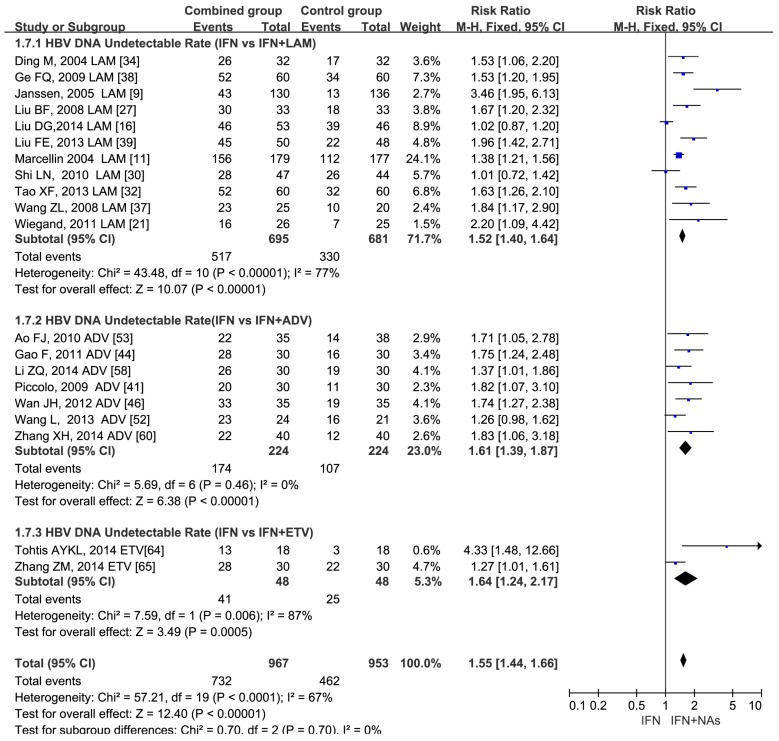
Forest plot of 1920 patients of 20 studies in HBV DNA undetectable rate between IFN and IFN plus NAs at week 48 treatment with a risk ratio 1.55 (95% CI: 1.44–1.66, *p* < 0.00001).

Compared with IFN monotherapy, combination therapy with NAs was superior in on-treatment virological and serological responses. At treatment week 24, combination group yielded a significant better virological responses than monotherapy (RR = 1.75, 95% CI: 1.56–1.96, *p* < 0.00001) (Supplementary Materials Figure S1). At treatment week 48, significant difference was also found in virological response which was sustained in the combination therapy (RR = 1.55, 95% CI: 1.44–1.66, *p* < 0.00001) (Figure 2).

**Figure 3:**
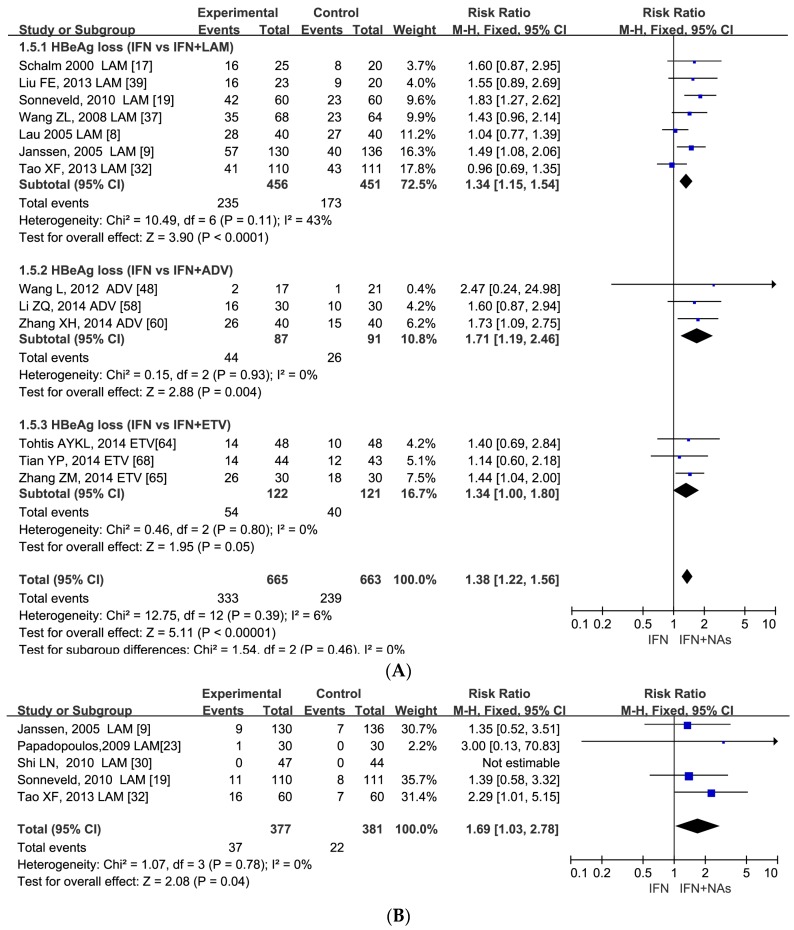
Forest plots of 13 studies including 1328 patients in HBeAg loss rate (RR = 1.38, 95% CI: 1.22–1.56, *p* < 0.00001) (**A**) and HBsAg loss rate in five studies including 758 patients between IFN and IFN plus NAs at week 48 treatment (RR = 1.69, 95% CI: 1.03–2.78, *p* = 0.04) (**B**).

At treatment week 24, higher HBeAg rate was observed in patients on combination therapy than monotherapy (RR = 1.45, 95% CI: 1.17–1.79, *p* = 0.0006) (Supplementary Materials Figure S2). At treatment week 48, the trend was similar (RR = 1.38, 95% CI: 1.22–1.56, *p* < 0.00001). In addition, a significant difference was also found in HBsAg loss rate (RR = 1.69, 95% CI: 1.03–2.78, *p* = 0.04) (Figure 3).

### 3.3. Similar Virological and Serological Responses in Different NAs Combination Therapies at Week 24 and 48 of Treatment

In virological response, studies including 1009 patients were retrieved in the analysis at week 24. Different NAs had similar responses, ETV combination therapy (RR = 1.76, 95% CI: 1.28–2.42), ADV combination therapy (RR = 1.71, 95% CI: 1.45–2.01) and LAM combination therapy (RR = 1.81, 95% CI: 1.50–2.18) (Supplementary Materials Figure S1). At week 48, the RRs of the three different NAs were: 1.64 (95% CI: 1.24–2.17) for ETV combination therapy; 1.61 (95% CI: 1.39–1.87) for ADV combination therapy; 1.52 (95% CI: 1.40–1.64) for LAM combination therapy, respectively (Figure 2).

In serological response, studies including 567 patients were retrieved in the analysis of HBeAg loss rate at week 24. The three different NAs also had a similar response. ETV combination therapy (RR = 1.52, 95% CI: 1.10–2.08), ADV combination therapy (RR = 1.43, 95% CI: 1.03–1.97) and LAM combination therapy (RR = 1.35, 95% CI: 0.77–2.38) (Supplementary Materials Figure S2). At week 48, the three different NAs had the following RRs: 1.34 (95% CI: 1.00–1.80) for ETV combination therapy; 1.71 (95% CI: 1.19–2.46) for ADV combination therapy; 1.34 (95% CI: 1.15–1.54) for LAM combination therapy, respectively (Figure 3).

### 3.4. Higher HBV DNA Undetectable Rate in NAs Combined with IFN Therapy at the End of Follow-Up of 24 –52 Weeks

**Figure 4:**
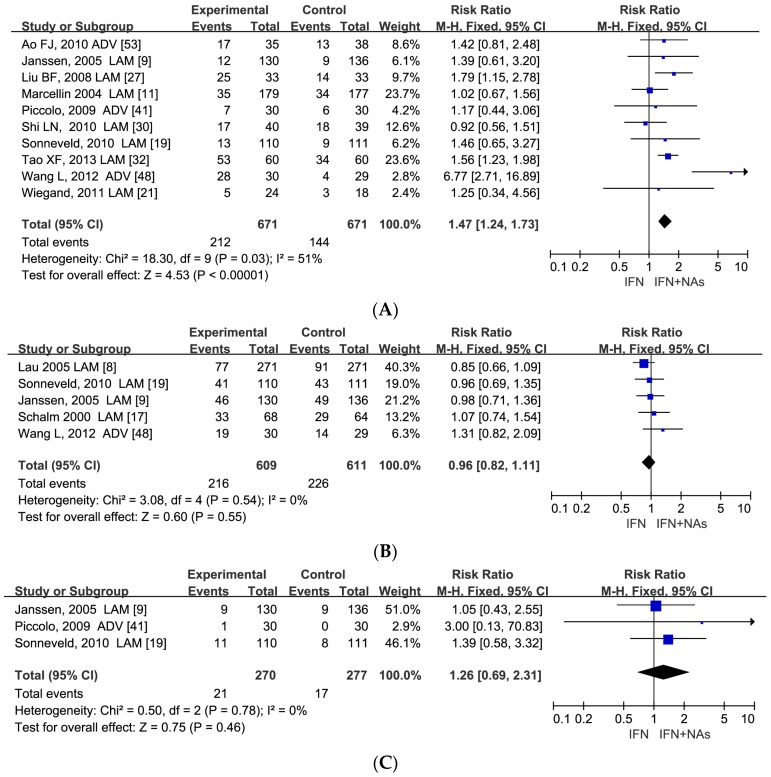
Forest plots of 10 studies including 1342 patients in HBV DNA undetectable rate (RR = 1.47, 95% CI: 1.24–1.73, *p* < 0.00001) (**A**), and five studies including 1220 patients in HBeAg loss rate (RR = 0.96, 95% CI: 0.82–1.11, *p* = 0.55) (**B**); and three studies including 547 patients in HBsAg loss rate (RR = 1.26, 95% CI: 0.69–2.31, *p* = 0.46) (**C**) between IFN and IFN plus NAs at the end of at least 24 weeks follow-up.

Ten studies with 1342 enrolled patients were included in the analysis of HBV DNA undetectable rate at the end of at least 24 weeks follow-up. NAs combination therapy was continuously superior to IFN monotherapy in HBV DNA undetectable rate with an RR of 1.47 (95% CI: 1.24–1.73, *p* < 0.00001). The virological response was sustained after withdrawal of anti-viral drugs for 24–52 weeks (Figure 4A).

### 3.5. Similar Serological Response between IFN Monotherapy and Combination Therapy at the Follow-Up 24–52 Weeks

After 24–52 weeks of follow-up, no significant difference was found between combination therapy and IFN monotherapy in either HBeAg loss rate or HBsAg loss rate, with the following RRs: 0.96 (95% CI: 0.82–1.11, *p* = 0.55) and 1.26 (95% CI: 0.69–2.31, *p* = 0.46), respectively (Figure 4B,C).

### 3.6. Publication Bias Test

**Figure 5:**
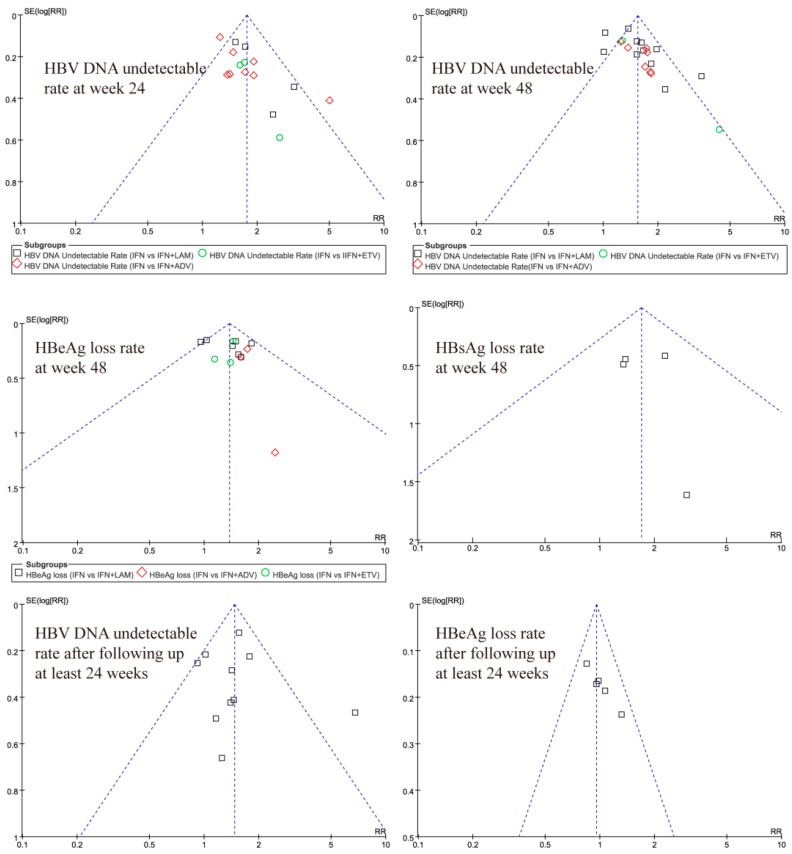
Funnel plots for relative risks of IFN and IFN plus NAs in virological and serological responses at different treatment periods.

The funnel plots resembled a symmetrical inverted funnel and no significant publication bias was detected among the individual studies of meta-analysis (Figure 5). Our assessment of the quality of the included studies is summarized in Supplementary Materials Table S1. Only five studies were considered to be of good overall quality, eighteen were assessed to be of fair quality, whilst the remainder were considered poor.

## 4. Discussion

This meta-analysis demonstrated that different NAs combined with IFN had similar virological and serological responses at week 24 and 48 of treatment. Furthermore, compared with IFN mono-therapy, IFN combined with NAs gave a better sustained virological suppression after the cessation of treatment.

Though previous meta-analyses had also shown that IFN combined with NAs gave a better HBV DNA suppression at the end of treatment, the sustained benefit of the combination therapy is still an issue for discussion. One meta-analysis showed that at the end of at least 24 weeks follow-up, IFN plus ADV resulted in better virological suppression [13], whereas lamivudine combination therapy was likely to be equally or less efficacious than pegylated interferon monotherapy [18]. As for ETV sustained virological response was not addressed in meta-analysis [69], in the current analysis, we compared sustained virological response of IFN with or without NAs (ETV, ADV, LAM) and found that combination therapies were more efficacious at least 24 weeks after the cessation of treatment.

In this study, we also carried out sub-analysis for the effectiveness of IFN combined with different NAs, including ADV and LAM which is widely used in China, as well as ETV which is recommended by WHO [70]. We found that at treatment week 48, ETV combination therapy yield slightly better virological response than other combination therapies. These results were not surprising, since ETV is of higher potency and suffers from less resistance issues than ADV and LAM.

There were several limitations of this study. First, there were limited randomized controlled trial studies which included ETV combination therapy partly because it was launched later than ADV and LAM. Consequently, we could not compare the sustained efficacy among different NAs combination therapy. Secondly, we only analyzed the virological and serological responses in this meta-analysis, because most studies had not reported histological improvement. Lastly, only a few studies included in the current meta-analysis were of high quality, although the publication bias was minimal.

## 5. Conclusions

This meta-analysis demonstrated that a better efficacy of NAs combination therapy than IFN monotherapy in virological and serological responses at the end of treatment. However, at the end of follow-up, only HBV DNA undetectable rate was superior in combination therapy. Therefore, in clinically practice, the benefits of combination therapy should be weighed against the higher cost.

## Supplementary Materials

The following are available online at www.mdpi.com/1660-4601/13/7/730/s1, Table S1: Characteristics of the 56 included trials, Figure S1: Forest plot of risk ratio (RR) in 1009 patients of 15 studies in HBV DNA undetectable rate between IFN and IFN plus NAs at week 24 treatment, Figure S2: Forest plot of risk ratio (RR) in 567 patients of nine studies in HBeAg loss rate between IFN and IFN plus NAs at week 24 treatment.
