# Supplementary material for: A Meta-Analysis of the Efficacy of Interferon Monotherapy or Combined with Different Nucleos(t)ide Analogues for Chronic Hepatitis B
Source: Int J Environ Res Public Health. 2016 Jul 21;13(7):730. doi: 10.3390/ijerph13070730 (PMC4962271; doi:10.3390/ijerph13070730)
Supplement: Supplementary file 1 [file ijerph-13-00730-s001.pdf]

Supplementary Materials: A Meta-Analysis of the Efficacy of Interferon Mono-Therapy or Combined with Different Nucleos(t)ide Analogues for Chronic Hepatitis B

Jialing Zhou, Xiaoning Wu, Wei Wei, Hong You, Jidong Jia and Yuanyuan Kong *

Table S1. Characteristics of the 56 included trials.

Authors (First)	Publish Year	Study Design	Treatment	No. (Combined)	No. (IFN)	No. (NAs)	No. (Total)	HBeAg (+/−)	Treatment Time (Week/Month)	Follow-Up (Week/Month)	Jadad Score
Lau, G.K. et al. [1]	2005	RCT	Lam + Peg IFN-α 2a	208	214	215	637	+	48 w	24 w	5
Janssen, H.L. et al. [2]	2005	RCT	Lam + Peg IFN-α 2b	130	136	0	266	+	52 w	26 w	7
Marcellin, P. et al. [3]	2004	RCT	Lam + Peg IFN-α 2a	179	177	181	537	+	48 w	24 w	6
Piratvisuth, T. et al. [4]	2008	RCT	Lam + Peg IFN-α 2a	350	446	344	1140	+/−	48 w	24 w	4
Liu, D.G. et al. [5]	2014	RCT	Lam + IFN	58	65	63	186	+	12 mon	6 mon	3
Schalm, S.W. et al. [6]	2000	RCT	Lam + α-IFN	75	69	82	151	+/−	52 w	12 w	6
Sonneveld, M.J. [7]	2010	RCT	Lam + Peg IFN-α 2b	110	111	0	221	+	52 w	26 w	4
Kaymakoglu, S. et al. [8]	2007	RCT	Lam + Peg IFN-α 2b	29	19	0	48	−	48 w	24 w	4
Wiegand, J. et al. [9]	2011	RCT	Lam + Peg IFN-α 2b	32	27	0	59	+/−	36 w	24 w	5
Ayaz, C. et al. [10]	2006	RCT	Lam + α-IFN	31	33	0	64	+	1/3/6 mon	6 mon	3
Papadopoulos, V.P. et al. [11]	2009	RCT	Lam + Peg IFN-α 2b	88	35	0	123	−	48 w	24 w	3
Yang, J. et al. [12]	2003	RCT	Lam + IFN-1b	40	33	39	112	+/−	1/3/6 mon	None	3
Ding, X.H. et al. [13]	2003	RCT	Lam + α-IFN	30	36	22	88	+	6 mon	13 w	2
Peng, J. et al. [14]	2007	RCT	Lam + α-IFN	24	18	21	63	+	26 w	None	2
Liu, B.F. et al. [15]	2008	RCT	Lam + IFN-α 2b	33	33	0	66	+	12 mon	26 w/52 w	2
Ding, N.L. et al. [16]	2010	RCT	Lam + IFN-α 2b	50	50	50	150	+	1/3/6/12 mon	None	2
Guo, B.Y. et al. [17]	2010	RCT	Lam + α-IFN	54	53	53	160	+	6/12 mon	None	2
Shi, L.N. [18]	2010	RCT	Lam + Peg IFN-α 2a	47	44	0	94	−	48 w	24 w	3
Huang, J.F. et al. [19]	2012	RCT	Lam + IFN-α 1b	62	49	0	111	+	3/6/9/12/18 mon	None	3
Tao, X.F. et al. [20]	2013	RCT	Lam + IFN-α 2a	60	60	0	120	+	12/24/52 w	26w	2
Chen, A.P. et al. [21]	2013	RCT	Lam + IFN-α 2b	72	72	0	144	+	24/48 w	None	2
Ding, M. et al. [22]	2003	RCT	Lam + α-IFN	32	32	32	96	+	52 w	26 w	3
Zhang, Z.L. et al. [23]	2005	RCT	Lam + IFN-α 2a	50	50	50	150	+	3/6 mon	26 w	2
Liu, Z.G. et al. [24]	2006	RCT	Lam + IFN	40	30	0	70	+/−	26 w	None	2
Wang, Z.L. et al. [25]	2008	RCT	Lam + α-IFN	25	20	25	70	+	3/6/12 mon	None	2
Ge, F.Q. et al. [26]	2009	RCT	Lam + IFN-α 2b	60	60	60	180	+	3/6/12 mon	None	2
Liu, F.E. et al. [27]	2013	RCT	Lam + IFN-α 1b	50	48	52	150	+	1/3/6/12 mon	None	3
Wu, J.S. et al. [28]	2013	RCT	Lam + IFN-α 2a	40	40	0	80	−	48 w	24 w	2
Piccolo, P. et al. [29]	2009	RCT	ADV + Peg IFN-α 2b	30	30	0	60	−	48 w	24 w	3
Li, Y.B. et al. [30]	2013	RCT	ADV + Peg IFN-α 2a	17	21	22	60	+	48 w	None	2

Table S1. *Cont.*

Ding, W.M. [31]	2010	RCT	ADV + Peg IFN-α 2a	22	20	23	65	+	48 w	None	2
Gao, F. et al. [32]	2011	RCT	ADV + Peg IFN-α 2b	30	30	30	90	+	12/24/48 w	None	2
Li, Z. [33]	2012	RCT	ADV + Peg IFN-α 2a	18	19	0	37	+	24/48 w	None	2
Wan, J.H. et al. [34]	2012	RCT	ADV + Peg IFN-α 2a	35	35	35	105	+	48 w	None	2
Wu, S.H. [35]	2010	RCT	ADV + Recombinant human IFN –α b	43	43	0	86	+	3/6 mon	None	2
Wang, L. et al. [36]	2012	RCT	ADV + Peg IFN-α 2b	30	29	31	90	+	12/24/48 w	24 w	3
Lai, D.Q. [37]	2013	RCT	ADV+ Peg IFN	42	42	0	84	+	48 w	None	2
Zhang, Q. [38]	2013	RCT	ADV + Peg IFN-α 2a	122	122	0	244	none	12 mon	None	2
Ji, M.F. [39]	2013	RCT	ADV + IFN	30	30	30	90	+	12/24/48 w	None	2
Wang, L. et al. [40]	2013	RCT	ADV + Peg IFN-α 2a	26	22	0	48	−	12/24/48 w	None	2
Wang, J.P. [41]	2014	RCT	ADV + Recombinant human IFN-α b	38	38	0	76	+	24 w	None	2
Hu, Y.S. et al. [42]	2009	RCT	ADV + Peg IFN-α	15	15	15	45	+	6 mon	None	3
Ao, F.J. et al. [43]	2010	RCT	ADV + Peg IFN-α 2a	40	40	40	120	+	24/48 w	48 w	3
Xu, T.M. et al. [44]	2012	RCT	ADV + Peg IFN-α 2b	42	40	0	82	+	48 w	None	2
Yang, Y.R. et al. [45]	2012	RCT	ADV + Peg IFN-α 2a	61	61	0	122	+	12/48 w	None	2
Li, Z.Q. et al. [46]	2014	RCT	ADV + IFN	30	30	30	90	+	48 w	0	2
Peng, F.J. et al. [47]	2014	RCT	ADV + IFN-α 2b	50	50	0	100	+	24 w	0	2
Zhang, X.H. et al. [48]	2014	RCT	ADV + Peg IFN-α 2a	40	40	0	80	none	48 w	0	2
Li, W.B. et al. [49]	2008	RCT	ADV + Peg IFN-α 2a	43	44	0	86	+	48 w	None	3
Chen, Y.S. et al. [50]	2013	RCT	ETV + IFN-α b	33	32	0	65	+	12/24/48 w	None	2
Zeng, W. et al. [51]	2013	RCT	ETV + Peg IFN-α a	20	20	20	60	+	12/24 w	None	2
Tohti, A.Y.K.L. [52]	2014	RCT	ETV + Peg IFN-α 2a	36	18	0	36	none	48 w	0	3
Zhang, Z.M. et al. [53]	2014	RCT	ETV + IFN-α 2b	30	30	30	90	+	48 w	0	3
Li, Y. [54]	2011	RCT	ETV + IFN-α 2b	20	20	20	60	+	12/24/48 w	None	2
Li, J. et al. [55]	2012	RCT	ETV + Peg IFN-α	44	43	0	87	+	12/24/48 w	None	2
Tian, Y.P. [56]	2014	RCT	ETV + IFN-α	44	43	51	138	+/−	12/24 w	None	2

Study or Subgroup	Experimental Events	Experimental Total	Control Events	Control Total	Weight	Risk Ratio M-H, Fixed, 95% CI
**4.1.1 HBV DNA Undetectable Rate (IFN vs IFN+LAM)**						
Ge FQ, 2009 LAM [26]	50	60	33	60	15.9%	1.52 [1.17, 1.96]
Peng J, 2007 LAM [14]	13	24	4	18	2.2%	2.44 [0.95, 6.24]
Tao XF, 2013 LAM [20]	48	60	28	60	13.5%	1.71 [1.27, 2.31]
Wang ZL, 2008 LAM [25]	24	25	6	20	3.2%	3.20 [1.63, 6.28]
**Subtotal (95% CI)**		**169**		**158**	**34.8%**	**1.81 [1.50, 2.18]**
Total events	135		71			
Heterogeneity: Chi² = 5.09, df = 3 (P = 0.17); I² = 41%						
Test for overall effect: Z = 6.13 (P < 0.00001)						
**4.1.2 HBV DNA Undetectable Rate (IFN vs IFN+ADV)**						
Ao FJ, 2010 ADV [43]	17	35	13	38	6.0%	1.42 [0.81, 2.48]
Gao F, 2011 ADV [32]	25	30	13	30	6.3%	1.92 [1.24, 2.98]
Hu YS, 2009 ADV [42]	11	15	8	15	3.8%	1.38 [0.78, 2.41]
Li ZQ, 2014 ADV [46]	19	30	11	30	5.3%	1.73 [1.00, 2.97]
Peng FJ, 2014 ADV [47]	23	48	12	48	5.8%	1.92 [1.08, 3.40]
Wang JP, 2014 ADV [41]	35	38	28	38	13.5%	1.25 [1.01, 1.54]
Wang L, 2013 ADV [40]	22	24	13	21	6.7%	1.48 [1.04, 2.12]
Wang L, 2012 ADV [36]	26	30	5	29	2.4%	5.03 [2.24, 11.30]
**Subtotal (95% CI)**		**250**		**249**	**49.8%**	**1.71 [1.45, 2.01]**
Total events	178		103			
Heterogeneity: Chi² = 17.28, df = 7 (P = 0.02); I² = 59%						
Test for overall effect: Z = 6.51 (P < 0.00001)						
**4.1.3 HBV DNA Undetectable Rate (IFN vs IIFN+ETV)**						
Tian YP, 2014 ETV [56]	28	44	16	43	7.8%	1.71 [1.09, 2.68]
Tohti AYKL, 2014 ETV [52]	8	18	3	18	1.4%	2.67 [0.84, 8.46]
Zhang ZM, 2014 ETV [53]	21	30	13	30	6.3%	1.62 [1.01, 2.59]
**Subtotal (95% CI)**		**92**		**91**	**15.5%**	**1.76 [1.28, 2.42]**
Total events	57		32			
Heterogeneity: Chi² = 0.64, df = 2 (P = 0.73); I² = 0%						
Test for overall effect: Z = 3.51 (P = 0.0005)						
**Total (95% CI)**		**511**		**498**	**100.0%**	**1.75 [1.56, 1.96]**
Total events	370		206			
Heterogeneity: Chi² = 24.08, df = 14 (P = 0.04); I² = 42%						
Test for overall effect: Z = 9.57 (P < 0.00001)						
Test for subgroup differences: Chi² = 0.19, df = 2 (P = 0.91), I² = 0%						

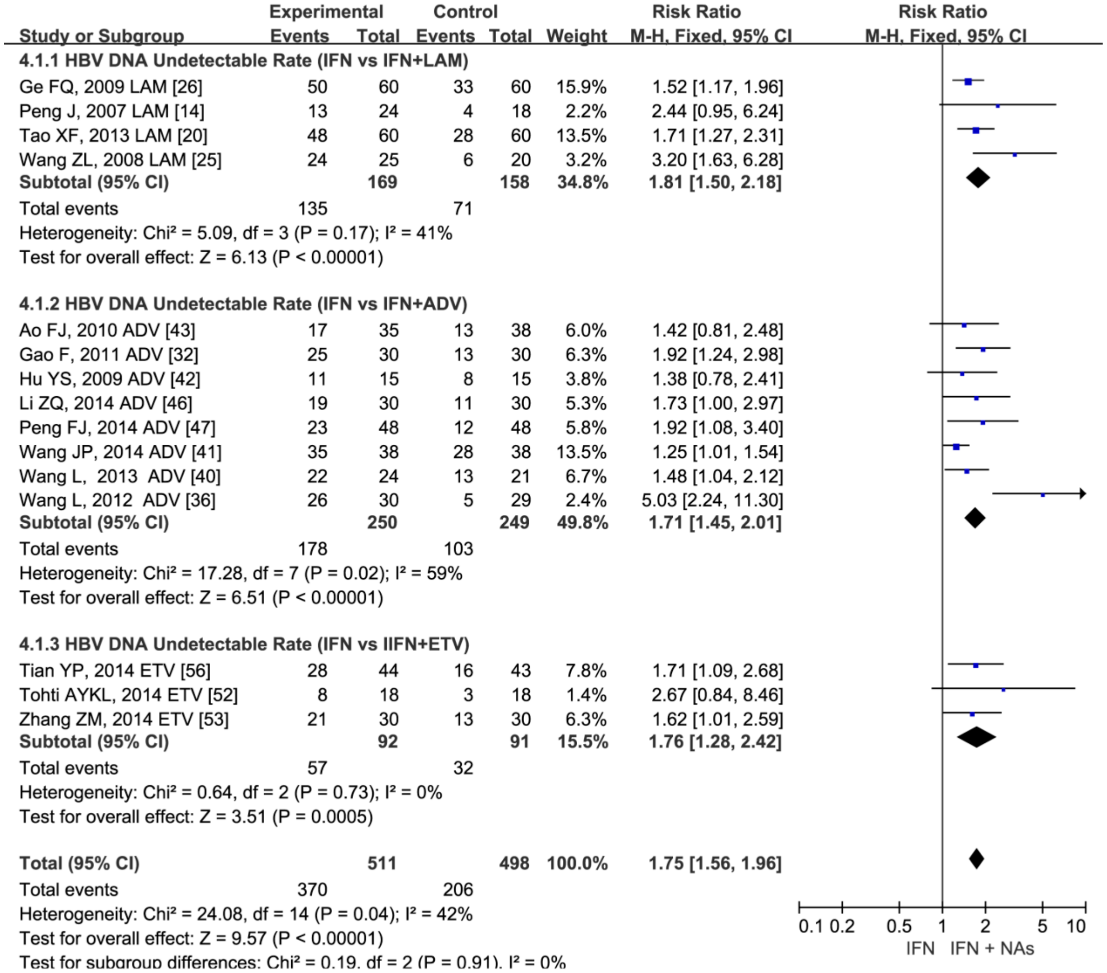

Figure S1. Forest plot of risk ratio (RR) in 1009 patients of 15 studies in HBV DNA undetectable rate between IFN and IFN plus NAs at week 24 treatment.

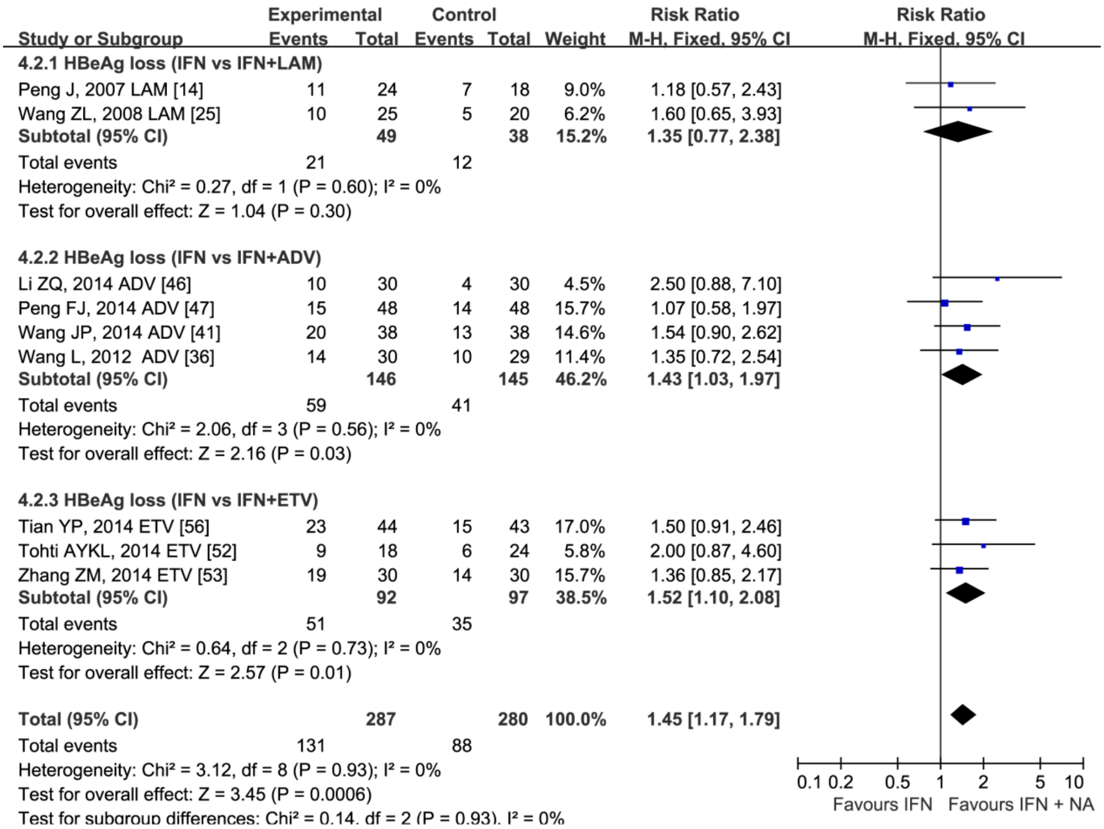

Figure S2. Forest plot of risk ratio (RR) in 567 patients of nine studies in HBeAg loss rate between IFN and IFN plus NAs at week 24 treatment.

References

1. Lau, G.K.; Piratvisuth, T.; Luo, K.X.; Marcellin, P.; Thongsawat, S.; Cooksley, G.; Gane, E.; Fried, M.W.; Chow, W.C.; Paik, S.W.; et al. Peg interferon alfa-2a, lamivudine, and the combination for HBeAg-positive chronic hepatitis B. *N. Engl. J. Med*. **2005**, *352*, 2682–2695.
2. Janssen, H.L.; van Zonneveld, M.; Senturk, H.; Zeuzem, S.; Akarca, U.S.; Cakaloglu, Y.; Simon, C.; So, T.M.; Gerken, G.; de Man, R.A.; et al. Pegylated interferon alfa-2b alone or in combination with lamivudine for HBeAg-positive chronic hepatitis B: A randomized trial. *Lancet* **2004**, *365*, 123–129.
3. Marcellin, P.; Lau, G.K.; Bonino, F.; Farci, P.; Hadziyannis, S.; Jin, R.; Lu, Z.M.; Piratvisuth, T.; Germanidis, G.; Yurdaydin, C.; et al. Peg interferon alfa-2a alone, lamivudine alone, and the two in combination in patients with HBeAg-negative chronic hepatitis B. *N. Engl. J. Med*. **2004**, *351*, 1206–1217.
4. Piratvisuth, T.; Lau, G.; Chao, Y.C.; Jin, R.; Chutaputti, A.; Zhang, Q.B.; Tanwandee, T.; Button, P.; Popescu, M. Sustained response to peg-interferon alfa-2a (40 kD) with or without lamivudine in Asian patients with HBeAg-positive and HBeAg-negative chronic hepatitis B. *Hepatol. Int*. **2008**, *2*, 102–110.
5. Liu, D.G. Efficacy of antiviral therapy in patients with chronic hepatitis B. *Guide China Med.* **2014**, *12*, 134–135. (In Chinese)
6. Schalm, S.W.; Heathcote, J.; Cianciara, J.; Farrell, G.; Sherman, M.; Willems, B.; Dhillon, A.; Moorat, A.; Barber, J.; Gray, D.F. Lamivudine and alpha interferon combination treatment of patients with chronic hepatitis B infection: A randomized trial. *Gut* **2000**, *46*, 562–568.
7. Sonneveld, M.J.; Rijckborst, V.; Boucher, C.A.; Hansen, B.E.; Janssen, H.L. Prediction of sustained response to peginterferon alfa-2b for hepatitis B e antigen-positive chronic hepatitis B using on-treatment hepatitis B surface antigen decline. *Hepatology* **2010**, *52*, 1251–1257.
8. Kaymakoglu, S.; Oguz, D.; Gur, G.; Gurel, S.; Tankurt, E.; Ersöz, G.; Ozenirler, S.; Kalayci, C.; Poturoglu, S.; Cakaloglu, Y.; et al. Pegylated interferon Alfa-2b mono-therapy and pegylated interferon alfa-2b plus lamivudine combination therapy for patients with hepatitis B virus E antigen-negative chronic hepatitis B. *Antimicrob. Agents Chemother*. **2007**, *51*, 3020–3022.

9. Wiegand, J.; Brosteanu, O.; Kullig, U.; Wiese, M.; Berr, F.; Maier, M.; Tillmann, H.L.; Schiefke, I. Quantification of HBsAg and HBV-DNA During therapy with peg-interferon alpha-2b plus lamivudine alpha-2b plus lamivudine and peg-interferon alpha-2b alone in a German chronic hepatitis B cohort. *Z Gastroenterol*. **2011**, *49*, 1463–1469.
10. Ayaz, C.; Celen, M.K.; Colak, H.; Hosoglu, S.; Geyik, M.F. Comparison of lamivudine and alpha-interferon combination with alpha-interferon alone in the treatment of HBeAg-positive chronic hepatitis B. *Indian J. Gastroenterol*. **2006**, *25*, 71–73.
11. Papadopoulos, V.P.; Chrysagis, D.N.; Protopapas, A.N.; Goulis, I.G.; Dimitriadis, G.T.; Mimidis, K.P. Peg-interferon alfa-2b as mono-therapy or in combination with lamivudine in patients with HBeAg-negative chronic hepatitis B: A randomized study. *Med. Sci. Monit*. **2009**, *15*, CR56–CR61.
12. Yang, J.; Zhang, L.S.; Zhao, X.K. Effect of single-using or combined-using lamivudine/interferon alpha-1b on serum markers in patients with hepatitis B. *Chin. J. Hepatol*. **2003**, *11*, 109–110. (In Chinese)
13. Ding, X.H.; Zhuang, L.; Sun, J.; Dong, J.L.; Chen, W.; Yang, Z.L.; Ge, H.J.; Yuan, H.X. Study on dynamic changes of hepatitis B virus during combination therapy of chronic hepatitis B with lamivudine and IFN. *Chin. J. Clini. Hepatol*. **2003**, *19*, 33–35. (In Chinese)
14. Peng, J.; Yang, J.W. Efficacy of interferon-α combined with lamivudine in the treatment of chronic hepatitis B. *Mod. Med. Health* **2007**, *23*, 1909–1910. (In Chinese)
15. Liu, B.F.; Tang, M.X.; Fan, B.C.; Wang, L.H. Clinical evaluation of lamivudine in combination with interferon a for chronic hepatitis B. *Chin. J. Gen. Pract*. **2008**, *6*, 946–947. (In Chinese)
16. Ding, N.L.; Zhu, X.; Wang, Y.P. Clinical observation on lamivudine combination interferon treatment patients with chronic hepatitis B. *China Med. Her.* **2010**, *7*, 47–48. (In Chinese)
17. Guo, B.Y.; Li, J.Z. Effect of lamivudine combined with alpha interferon with chronic HBeAg-positive hepatitis. *Med. J. Qilu* **2010**, *2*, 246–250. (In Chinese)
18. Shi, L.N. Comparison between pegylated interferon mono-therapy and pegylated interferon plus lamivudine combination therapy for efficacy and safety in HBeAg-negative patients. *Chin. Gen. Pract.* **2010**, *13*, 3867–3869. (In Chinese)
19. Huang, J.F.; Jin, R.; Liu, Z.H.; Li, H.W.; Xie, Y.M. The effect of recombinant human interferon α-1b combined with lamivudine on treatment HBeAg positive chronic hepatitis B. *Mod. Med. J. China* **2012**, *14*, 18–21. (In Chinese)
20. Tao, X.F.; Luo, X.Y.; Lou, L.Q.; Zhu, J.H.; Chen, Y.X.; Ye, W.W.; Wu, S.; Chen, X.Y. Efficacy of combination therapy with Peg IFNα-2a and lamivudine for treating HBeAg positive chronic hepatitis B patients. *Chin. J. Microecol*. **2013**, *25*, 1330–1332. (In Chinese)
21. Chen, A.P. Clinical efficiency analysis of lamivudine combination with recombinant human interferon α-2b in the treatment of 144 cases of patients with chronic hepatitis B. *J. Hainan Med. Univ*. **2013**, *19*, 209–211. (In Chinese)
22. Ding, M.; Jiang, S.Z.; Wang, J.H. The efficacy of lamivudine combined with interferon treatment of chronic hepatitis B. *Zhejiang Prev. Med*. **2003**, *15*, 67–68. (In Chinese)
23. Zhang, Z.L.; Yang, Z.G.; Liu, J.F. Clinical observation of 50 cases of a-interferon combined with lamivudine of chronic hepatitis B. *Ningxia Med. J*. **2005**, *27*, 199–200. (In Chinese)
24. Liu, Z.G.; Cao, M.G. The efficacy of lamivudine combination with interferon therapy in chronic hepatitis B. *Mod. J. Integr. Tradit. Chin. West. Med*. **2006**, *15*, 720–721. (In Chinese)
25. Wang, Z.L.; Jia, S.J.; Chen, J.H. Observation of lamivudine combined with interferon treatment of HBeAg-positive chronic hepatitis B patients. *Mod. J. Integr. Tradit. Chin. West. Med*. **2008**, *17*, 522–523. (In Chinese)
26. Ge, F.Q.; Xu, J.; Geng, J.H.; Huang, G.Q. Comparison of the efficacy of lamivudine in combination with or interferon alone in the treatment of chronic hepatitis B. *Chin. J. Misdiagnosis* **2009**, *9*, 8346–8347. (In Chinese)
27. Liu, F.E.; Xie, Y.L.; Li, X.J.; Lu, J; Liu, C.M.; Mo, M. Clinical study of lamivudine combined with interferon-l b treatment of chronic hepatitis B. *J. Chin. Phys.* **2013**, *15*, 920–923. (In Chinese)
28. Wu, J.S. Comparing the early efficacy and safety of Pegasys alone with Pegasys in combination with lamivudine in HBeAg-negative chronic hepatitis B patients. *Seek Med. Ask Med.* **2013**, *11*, 290–291. (In Chinese)
29. Piccolo, P.; Lenci, I.; Demelia, L.; Bandiera, F.; Piras, M.R.; Antonucci, G.; Nosotti, L.; Mari, T.; De Santis, A.; Ponti, M.L.; et al. A randomized controlled trial of pegylated interferon-alpha2a plus adefovir dipivoxil for hepatitis B e antigen-negative chronic hepatitis B. *Antivir. Ther*. **2009**, *14*, 1165–1174.

30. Li, Y.B.; Ma, G.Q.; Xu, L.; Zhang, X.Q.; Sui; H.L.; Ding, L. The clinical efficacy of peg-IFN α-2a combined with adefovir dipivoxil for HBeAg-positive chronic hepatitis B. *Chin. J. Clin. Infect. Dis*. **2008**, *1*, 168–170. (In Chinese)
31. Ding, W.M. The efficacy of Peg-IFN combined with adefovir dipivoxil treatment for 22 cases of chronic hepatitis B. *Chin. J. Mod. Drug Appl*. **2010**, *4*, 164–165. (In Chinese)
32. Gao, F.; Zhao, L.F. The efficacy of interferon a-2b combination adefovir or alone in HBeAg-positive chronic hepatitis B patients. *Natl. Med. Front. Chin.* **2011**, *6*, 28–29. (In Chinese)
33. Li, Z. The efficacy of alfa-2a 180 ug combined with adefovir dipivoxil in chronic hepatitis B. *World Latest Med. Inf.* **2012**, *12*, 267–268. (In Chinese)
34. Wan, J.H.; Liu, F.X. The efficacy of peg-interferon a-2a combined with adefovir dipivoxil in HBeAg-positive chronic hepatitis B patients. *Chin. Health Care Nutr.* **2012**, *22*, 4351–4352. (In Chinese)
35. Wu, S.H. The clinical studies of adefovir dipivoxil combined with recombinant human interferon a-2b in chronic hepatitis B treatment. *Med. Innov. Chin.* **2010**, *7*, 65–66. (In Chinese)
36. Wang, L.; Zhang, X.Y.; Yu, H.Y.; Yang, J.M.; Cao, X.G.; Ding, Q.Y.; Pan, J. Clinical observation of interferon α-2b combined with adefovir dipivoxil in HBeAg positive chronic hepatitis B. *Chin. J. Biochem. Pharm*. **2012**, *33*, 849–851. (In Chinese)
37. Lai, D.Q. Clinical observation of interferon plus adefovir dipivoxil therapy in chronic hepatitis B. *Med. Forum* **2013**, *17*, 1297–1298. (In Chinese)
38. Zhang, Q. Investigate the effect of 50 cases interferon α-2a combined with adefovir dipivoxil chronic hepatitis B. *Med. Inf.* **2013**, *26*, 149–150. (In Chinese)
39. Ji, M.F. The research of combination therapy with adefovir dpivoxil. *Northwest Pharm. J.* **2013**, *28*, 635–636. (In Chinese)
40. Wang, L.; Zhang, X.Y.; Tian, Y.L.; Zang, Z.D.; Zhu, X.Y.; Yin, W.W.; Fu, X.L. The efficacy of peg-interferon combined with adefovir dipivoxil for HBeAg-negative chronic hepatitis B. *Chin. Hepatol*. **2013**, *18*, 839–841. (In Chinese)
41. Wang, J.P. Clinical observation of 38 cases of interferon in combination with adefovir dipivoxil treatment chronic hepatitis B. *Chin. J. Mod. Drug Appl*. **2014**, *8*, 146–146. (In Chinese)
42. Hu, Y.S.; Li, H.Y. The analysis of the influence of combining adefovir dipivoxil with-IFN on the hepatitis B markers. *Chin. Pract. Med*. **2009**, *4*, 45–46. (In Chinese)
43. Ao, F.J.; Ma, W.M.; Zhou, P.P.; Zhou, D.Q.; Hu, Y.W.; He, Q.; Dai, W.; Xu, B.; Peng, Y.Z., Fan, Q.; et al. Comparison of efficacy and safety of pegylated interferon alfa-2a or adefovir dipivoxil mono-therapy with combination therapy in HBeAg positive chronic hepatitis B patients. *Chin. J. Infect. Dis*. **2010**, *28*, 214–217. (In Chinese)
44. Xu, T.M.; Tong, X.C. The short-term efficacy and safety of interferon alfa-2b in combination with adefovir dipivoxil in treatment of patients with HBeAg positive chronic hepatitis B. *J. Clin. Hepatol*. **2012**, *15*, 506–507. (In Chinese)
45. Yang, Y.R.; Li, H.; Jia, T.; Shen, L. The curative effect of polyethylene glycol (peg) Interferonalpha-2a combined with adefovir on HBeAg positive chronic hepatitis B. *J. Kunming Med. Univ.* **2012**, *22*, 106–108. (In Chinese)
46. Li, Z.Q.; Zhen, J.Q.; Zhao, M.C. Clinical efficacy of interferon combined with adefovir dipivoxil in treatment of chronic hepatitis B. *J. Bethune Med. Sci*. **2014**, *12*, 114–115. (In Chinese)
47. Peng, F.J.; Zhang, T.X.; Yuan, M.R.; Chen, F.S. Clinical observation of Interferon combined with adefovir dipivoxil in the treatment of patients with HBeAg (+) chronic hepatitis B. *Chin. Phamacist.* **2014**, *17*, 984–986. (In Chinese)
48. Zhang, X.H.; Yi, C.G. Clinical observation of adefovir dipivoxil and polyethylene glycol interferon alfa-2a in the treatment of chronic hepatitis B. *J. Mod. Med. Health* **2014**, *30*, 1637–1638. (In Chinese)
49. Li, W.B.; Ding, J.G.; Sun, Q.F.; Hong, L.; Fu, R.Q. Efficacy of pegylated interferon alpha-2a combined with adefovir dipivoxil in treatment of HBeAg positive chronic hepatitis B. *Chin. J. Nosocomiol.* **2013**, *23*, 1250–1251. (In Chinese)
50. Chen, Y.S.; Fei, X.Y.; Liu, W.T.; Shu, Q. The efficacy of 33 cases of interferon alpha-1b in combination with entecavir on HBeAg positive chronic hepatitis B patients. *J. Chin. Intern. Med*. **2013**, *30*, 42–43. (In Chinese)
51. Zeng, W.; Yuan, J.; Liu, Y.X.; Zhang, Y.; Li, S.Q.; Yao, S.M.; Lin, Y.M.; Chen, C.T.; Zhao, M.F.; Zhou, B.T.; et al. Efficacy of peg-interferon alpha-2a combined with entecavir on HBeAg positive chronic hepatitis B patients with high serum hepatitis B viral loads. *Chin. J. Exp. Clin. Virol.* **2013**, *27*, 115–118. (In Chinese)

52. Tohti, A.Y.K.L. Pegylated interferon alpha-2a combined with entecavir cure analysis of 18 cases of chronic hepatitis B. *World Latest Med. Inf.* **2014**, *14*, 150–151. (In Chinese)
53. Zhang, Z.M.; Ji, B.Y.; Li, L. Effect of entecavir combined with α-2b interferon on treatment of chronic hepatitis B. *J. Bengbu Med. Coll.* **2014**, *39*, 621–623. (In Chinese)
54. Li, Y. The efficacy of α-2b interferon combined with entecavir in patients with chronic hepatitis B. *China Pract. Med.* **2011**, *6*, 136–137. (In Chinese)
55. Li, J. The efficacy of early treatment of chronic hepatitis B of interferon combined with entecavir. *Chin. Hepatol.* **2012**, *17*, 714–715. (In Chinese)
56. Tian, Y.P. Clinical observation of IFN- and entecavir in treating chronic hepatitis B at the initial stage. *China Mod. Dr.* **2014**, *52*, 22–24. (In Chinese)

© 2016 by the authors; licensee MDPI, Basel, Switzerland. This article is an open access article distributed under the terms and conditions of the Creative Commons by Attribution (CC-BY) license (http://creativecommons.org/licenses/by/4.0/).
